# A reference low-pressure CO_2_ adsorption isotherm for zeolite 13X: results of an interlaboratory study

**DOI:** 10.1007/s10450-026-00701-3

**Published:** 2026-08-28

**Authors:** Huong Giang T. Nguyen, David Newton, Riaz Ahmad, Carsten Prinz, Mario Zorrilla-Valtierra, Krista S. Walton, Killian Gmyrek, Ronny Pini, Camille Petit, Marcus Lange, Jens Mӧllmer, Evelyn Fuhrmann, Katharina Volz, Garrett Taggart, Andreas Schreiber, Yuko Konishi, Kazuyuki Nakai, Christos Tampaxis, Theodore Steriotis, Hai Wang, Charles David Brewster, Mi Tian, Rajan Jagpal, Siyi Liu, George Neville, Sunanda Sain, Timothy Mays, Riccardo Rea, Enzo Mangano, Stefano Brandani, Maxwell Tsipoaka, Fateme Rezaei, Adrien Houzé, Nicolas Heymans, Guy De Weireld, Charles E. Holland, Armin D. Ebner, James A. Ritter

**Affiliations:** 1 National Institute of Standards and Technology, Gaithersburg, MD USA; 2 Anton Paar QuantaTec Inc, Boynton Beach, FL USA; 3 Federal Institute for Materials Research and Testing, Berlin, Germany; 4 Georgia Institute of Technology, Atlanta, GA USA; 5 Imperial College London, London, UK; 6 Institut für Nichtklassische Chemie, Leipzig, Germany; 7 Micromeritics GmbH, Unterschleißheim, Germany; 8 Microtrac Inc, York, PA USA; 9 Microtrac Retsch GmbH, Haan, Germany; 10 MicrotracBEL, Suminoe-ku, Osaka, Japan; 11 National Centre for Scientific Research “Demokritos”, Athens, Greece; 12 National Institute of Metrology, Beijing, China; 13 University of Bath, Bath, UK; 14 University of Edinburgh, Edinburgh, UK; 15 University of Miami, Coral Gables, FL USA; 16 University of Mons, Mons, Belgium; 17 University of South Carolina, Columbia, SC USA

**Keywords:** Zeolite 13X, CO_2_, Interlaboratory study, Research grade test material, Reference isotherm, VAMAS

## Abstract

This paper reports the results of an international interlaboratory study sponsored by the Versailles Project on Advanced Materials and Standards (VAMAS) and led by the National Institute of Standards and Technology (NIST) on the measurement of low-pressure CO_2_ adsorption isotherms at 25 °C on zeolite 13X (NIST research grade test material 10257). Eighteen laboratories participated in the study and contributed 21 datasets. From these data, a consensus reference isotherm, along with the 95% uncertainty interval (*U*_*k=2*_), were determined and reported. Results indicate the material is suitable for development into NIST reference material, which will become the first zeolitic reference material in beaded form at NIST.

**Supplementary Information:**

The online version contains supplementary material available at https://doi.org/10.1007/s10450-026-00701-3.

## Introduction

Solid adsorbent materials have great potential for gas separation [1], gas and water purification [2], gas storage [3, 4], and as industrial catalysts [5–8]. A key metric in the performance of an adsorbent is an adsorption isotherm, which determines the gas uptake as a function of equilibrium pressure at a constant temperature. To ensure reliable and reproducible data, the National Institute of Standards and Technology (NIST), in partnership with the Department of Energy’s Advanced Research Projects Agency (ARPA-E), started a program in 2014 to improve adsorption metrology through the development of measurement protocols, reference materials, and reference data. At the time, existing gas adsorption reference materials mainly provided reference data associated with low-pressure, cryogenic adsorption experiments for adsorbent characterization. Examples include characterization of micropore volume and median micropore width from Ar adsorption isotherm at 87 K on reference zeolites (BCR-704 and BCR-705) from the Federal Institute for Materials Research and Testing (BAM) in Germany, and characterization of Brunauer-Emmett-Teller surface area from N_2_ adsorption isotherms at 77 K on TiO_2_ nanomaterial (SRM 1898), silicon nitride (SRM 1900), and controlled pore glass with nominal pore diameters of 18 nm and 300 nm, (SRM 2206 and SRM 2207, respectively) from NIST.

The Facility for Adsorbent Characterization and Testing (FACT Lab) at NIST has since led three interlaboratory studies (ILSs) to provide reference adsorption isotherms for other gases and vapors of interests to the adsorption community using existing reference materials [9]. ILS1, ILS2, and ILS3, respectively, led to the determination of a reference high-pressure surface excess isotherm for adsorption of carbon dioxide on NIST RM 8852 (ammonium ZSM-5 zeolite) at 20 °C up to 4.5 MPa [10], a reference high-pressure surface excess isotherm for adsorption of methane on NIST RM 8850 (sodium zeolite Y) at 25 °C up to 7.5 MPa [11], and reference water vapor sorption isotherms on BAM-P109 (nanoporous carbon) at 25 °C as a function of relative pressure (P/P_0_) for pure water measurements and relative humidity, with N_2_ as the carrier gas [12]. Efforts to advance adsorption metrology were also underway at other National Metrology Institutes. Notably, BAM led an interlaboratory study under the Metrology for Advanced Hydrogen Storage Solutions (MefHySto) framework to evaluate pelleted ZIF-8, a zeolitic imidazolate framework, as a reference material for hydrogen cryoadsorption (77 K) up to 10 MPa [13].

Currently, no low-pressure CO_2_ adsorption reference standards (materials or data) exist from NIST or other organizations. Adsorption of ultralow CO_2_ concentration is relevant to applications, such as direct air capture (DAC) of the approximately 430 ppm CO_2_ in air to mitigate climate change [14, 15], CO_2_ scrubbing for inhabitable air in enclosed space (e.g. spaceships, submarines) [16], and specialized industrial processes that require high-purity gases or precisely controlled air composition. As such, the ability to fine tune the capture and release of CO_2_ has garnered much interest. Solid adsorbent to adsorb CO_2_ is one means for carbon capture, storage, and utilization by adjusting temperature and pressure [17]. Consequently, NIST developed a zeolitic Research-Grade Test Material (RGTM) [18] for ultralow CO_2_ adsorption measurements to enable instrument validation and interlaboratory comparison of data, ultimately to accelerate the commercialization of materials. This paper reports the results of an interlaboratory study (hereafter ILS4) organized through Technical Working Area (TWA) 39 on Solid Sorbents of the Versailles Project on Advanced Materials and Standards (VAMAS) to investigate low-pressure CO_2_ sorption isotherm at 25 °C on zeolite 13X beads.

The objectives of ILS4 are threefold: (1) provide low-pressure CO_2_ reference data; (2) provide a new zeolitic reference material at NIST in beaded form; and (3) determine the suitability of the material for future ILSs looking at gas mixtures.

The sorbent used in this study is RGTM 10257 (zeolite 13X). Zeolite 13X is a Faujasite (FAU) molecular sieve with application in air purification, water removal, and gas separation. This common zeolite was chosen because it is expected to be stable and exhibits a measurable quantity of CO_2_ uptake at DAC-relevant, ultra-dilute CO_2_ concentrations in the parts per million (µmol/mol) range allowing for reference adsorption data at as low as 1 Pa. This contrasts with ZSM-5 zeolite used in ILS1, which exhibits essentially no CO_2_ adsorption at 40 Pa. Consequently, while ILS1 also looked at CO_2_, it was focused on high-pressure CO_2_ adsorption isotherm and only provided reference adsorption values down to 1 kPa, below which few data points were presented. The beaded form of zeolite 13X was chosen because it offers benefits such as easier sample handling, recovery, and clean up after testing, while also reducing sample loss and dust generation compared with powder form. Furthermore, if suitable for development into a NIST reference material, RGTM 10257 will be the first beaded-form NIST zeolitic reference material and could offer a material platform for future ILSs focused on dynamic adsorption measurements, or breakthroughs, which may require the sample to be in pelletized, beaded or granular form. The mechanically robust beaded/granular structure allows more reproducible packing in the sample holder and supports good gas flow through the bed.

## Experimental and data analysis methods

### Methods

The ILS involved measurement of low-pressure CO_2_ sorption isotherms at 25 °C up to 100 kPa. Zeolite 13X 8–12 mesh (1.68 mm to 2.38 mm) beads were acquired from a commercial source. The material was further sieved to ensure bead particle size homogeneity to the stated 8–12 mesh size before riffling into glass vials with ≈ 11 g each. These units were distributed to the study participants and designated Research Grade Test Material (RGTM) 10257. While zeolite 13X is available from multiple vendors and in various mesh sizes, and it should not be implied that all commercially available zeolite 13X will have the same properties as reported here for RGTM 10257. While RGTM 10257 is currently available for ILSs only, once it is developed into a reference material, it will be available for purchase through NIST.

Eighteen laboratories from seventeen institutions participated in the ILS. The measurement capabilities of these laboratories included both manometric and gravimetric commercial instruments, the majority of which were dedicated low-pressure adsorption instruments.

The measurement protocol instructions for the ILS were minimal. The protocol specified a minimum purity of 99.999% for all gases used. The sample was to be outgassed in vacuum at room temperature, then heated at a rate of 1 °C/min to 350 °C and held at that temperature for 12 h under high vacuum with continuous pumping (using a turbomolecular pump). If the outgassing was performed in a separate manifold or equipment, exposure to air was to be avoided when transferring to the analysis port. Fifty-five equilibrium pressure points from 2 Pa to 100 kPa were recommended to obtain a well-defined adsorption isotherm. However, if an instrument could not achieve the lowest recommended pressure points, data was to be recorded starting at the lowest pressure point the instrument could achieve. Measurements were to be conducted at 25 °C. It was requested that a complete isotherm, with adsorption and desorption, be measured on two separate aliquots, activated individually, two cycles each, resulting in a total of four isotherms. It was recommended to perform a blank run (i.e., an isotherm in the absence of the adsorbent) to subtract from the isotherm measured with the adsorbent present [19]. Participants were asked to submit an experimental report, which detailed their experimental procedures and data processing steps and to submit the isotherms in units of millimoles of CO_2_ adsorbed per gram of zeolite 13X (mmol/g). Details of the experimental parameters and procedures employed by each of the study participants can be found in Table 1. Certain commercial items are identified in this paper. This identification does not imply recommendation by NIST, nor does it imply that these items are the best available for the purposes described.

### Data submission

Twenty-one datasets (DS1-DS21) were submitted. A dataset is composed of four isotherms from two aliquots of sorbent, two cycles each. DS7 measured four different aliquots once rather than two cycles each of two aliquots. The submitted isotherms consist of adsorption and then desorption with the exception of DS9 and DS10, which did not provide the desorption.

### Dataset display

The plots shown in the figures in the text include all 4 isotherms for each dataset unless otherwise noted. The individual plots for each dataset can be found in the Supplementary Information (Figures S1-S8). The statistical analyses involved all four adsorption isotherms from all datasets that were determined to be acceptable (see *Results and Discussion* section for determination of outliers/omitted datasets). The number of each dataset is random and does not correspond to the listing of authors.

### Determination of datasets for omission

The 21 as-submitted datasets were evaluated based on their reported experimental details such as outgassing conditions and sample handling, zeolite mass loss from outgassing, and the blank and its application or lack thereof. From the technical evaluation, four datasets were flagged for omission (DS9, D17, DS19, and DS20) and participants who submitted those datasets were notified. DS1 self-reported issue with the calibration of their pressure transducer, so DS1 was also omitted. Unlike in previous ILSs, while these participants were given the opportunity to remeasure or reprocess the submitted isotherms to identify pitfalls, remeasured or reprocessed datasets in this ILS were not included in the determination of the reference isotherm. Two datasets (DS1, DS17) were remeasured and three datasets (DS9, DS19, and DS20) were reprocessed by NIST for blank/mass issues.

### Reference isotherm determination

The isotherm data consist of uptake as a function of pressure collected from 18 participating labs. Due to the overall highly nonlinear nature of the uptake function, the datasets were analyzed on a log_10_-log_10_ scale so that the functional form is easier to model with closed-form functions. Furthermore, given the data were grouped by dataset, aliquot, and cycle, hierarchical modeling was used to account for the correlation within the same cycles, aliquots, and datasets. Specifically, the model included additive random effect terms for aliquot and cycle on the log_10_-log_10_ scale. After removing datasets with identified issues (DS1, DS9, DS17, DS19, and DS20), a segmented regression model was used to fit the mean function of the log_10_-log_10_ scale, where the function is linear up until a point, which was set to be log(*P/*kPa) = -1.5 (or *P* = 0.0316 kPa). For log(*P/*kPa) greater than – 1.5, the function is a 5th order polynomial. The regression function was also constrained to be “smooth” (continuous first derivative) at log(*P/*kPa) = -1.5. The models were fit using the *lmer* function from the *lme4 R* package. To account for correlations within labs and experimental runs, random intercept terms were included for lab and experimental run. The coefficients can be found in Table 2. The back-transformed, raw scale fit function is given by Eq. (1) for *P* ≤ 0.0316 kPa, and Eq. (2) for 0.0316 kPa ≤ *P* ≤ 100 kPa,

1 $$\:\frac{{n}_{ex,ref}\left(P\right)}{\left(\mathrm{m}\mathrm{m}\mathrm{o}\mathrm{l}\mathrm{/g}\right)}={10}^{a+b\:\mathrm{l}\mathrm{o}{\mathrm{g}}_{10}\left(\frac{P}{k\mathrm{P}\mathrm{a}}\right)}$$

2 $$\begin{aligned} &\:\frac{{n}_{ex,ref}\left(P\right)}{\left(\mathrm{m}\mathrm{m}\mathrm{o}\mathrm{l}\mathrm{/g}\right)}\\&={10}^{a+b\:\mathrm{l}\mathrm{o}{\mathrm{g}}_{10}\left(\frac{P}{\mathrm{k}\mathrm{P}\mathrm{a}}\right)+c\:\left({{(\mathrm{log}}_{10}\left(\frac{P}{\mathrm{k}\mathrm{P}\mathrm{a}}\right)+1.5)}^{2}\right)\:+\:d\:\left({{(\mathrm{log}}_{10}\left(\frac{P}{\mathrm{k}\mathrm{P}\mathrm{a}}\right)+1.5)}^{3}\right)+e\:\left({{(\mathrm{log}}_{10}\left(\frac{P}{\mathrm{k}\mathrm{P}\mathrm{a}}\right)+1.5)}^{4}\right)+f\:\left({{(\mathrm{log}}_{10}\left(\frac{P}{\mathrm{k}\mathrm{P}\mathrm{a}}\right)+1.5)}^{5}\right)} \end{aligned} $$

where *n*_*ex, ref*_ = surface excess uptake (mmol/g), *P* = equilibrium pressure (kPa), *a*, *b*, *c*, *d*, *e*, and *f* are empirical parameters shown in Table 2. Quantile regression as implemented in the *quantreg R* package was used to estimate a 95% uncertainty interval (*U*_*k=2*_) for the fitted functions. For Eq. (1), where *P* ≤ 0.0316 kPa, estimates for upper and lower bounds at the 95% level are given by linear quantile regression functions at the 0.975 and 0.025 levels provided. For 0.0316 kPa ≤ *P* ≤ 100 kPa, estimates for upper and lower bounds at the 95% level (*U*_*k=2*_) are given by a constant value. All fit functions were selected because they replicated the form of the measured isotherms, but no physical significance should be associated with the functions or their parameters.

### Sources of uncertainty

To investigate the relative contributions of the various sources of uncertainty within the dataset (lab, aliquot, cycle, measurement variability), a hierarchical model of each of these components was fit to the residuals of the reference isotherm determination. Since the behavior of the recorded observations changes meaningfully above the transition point *P* = 0.0316 kPa, this modeling was only applied to residuals above this threshold, so that excessive variation near the lower pressures does not dominate the model fits. For 0.0316 kPa ≤ *P* ≤ 100 kPa, the hierarchical model estimated the relative contributions of uncertainty, *u*, given in Table 3.

## Results and discussions

### As-submitted datasets

**Fig. 1:**
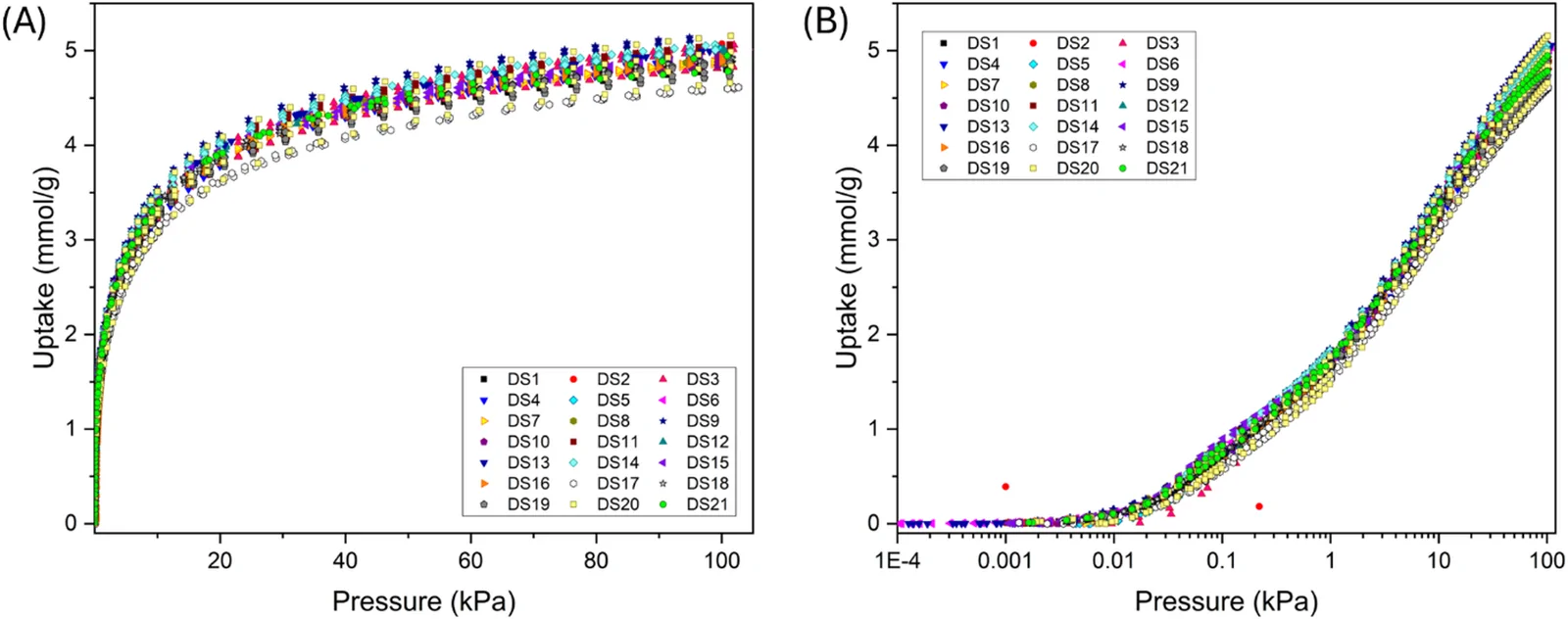
As-submitted surface excess CO_2_ adsorption isotherms at 25 °C for zeolite 13X up to 100 kPa plotted in (**A**) linear and (**B**) semi-log scales (For individual datasets, see Figures S1-S8)

In total, 21 low-pressure CO_2_/zeolite 13X datasets were submitted by 18 labs, three of whom submitted 2 datasets each using two different instruments in their respective labs. With the CO_2_ adsorption and desorption isotherms overlapping at 25 °C on 13X, only the adsorption isotherms of the as-submitted datasets are plotted together in Fig. 1 (See Figures S1-S8, for individual isotherms with adsorption and desorption). It should be noted that for DS8, desorption deviates from adsorption below 0.1 kPa. The deviation in the desorption can be attributed to the dynamic measurement and much greater uncertainties at these low-pressure levels. The low-pressure CO_2_ adsorption isotherms on zeolite 13X at 25 °C appear to follow a Type I isotherm shape. See Figure S9 for *P/P*_*0*_ scale and a measurement at extended range up to 0.6 *P/P*_*0*_, where *P*_*0*_ = 6.43 MPa using Span and Wagner equation of state [20].

### Identification of pitfalls in the omitted datasets

From observation of Fig. 1, DS17 stood out as having the lowest adsorption capacity, while DS9 exhibited slightly higher adsorption capacity compared to the other datasets. Further evaluation of these two datasets found the zeolite 13X sample mass loss from outgassing for DS17 to be unusually low and that for DS9 to be slightly high as shown in Figure S10. Given that DS9 and DS17 had mass losses outside of the 95% expanded uncertainty of the average mass loss of 21.4%, both datasets were considered to be outliers and not included in the determination of the reference isotherm. It is interesting to note that the participant who submitted DS9 submitted two datasets, and once DS9 was renormalized with the average mass loss from the second dataset, it agreed well with the other datasets, suggesting a mass measurement issue. While this dataset could be improved with reprocessing, we did not include any remeasured or reprocessed datasets in the determination of the reference data in this study. For DS17, the participant indicated that the dry (outgassed) sample mass was not weighed until after the adsorption isotherm measurements, after which the sample was backfilled with CO_2_ (which the sample adsorbs), causing a higher “dry” sample mass, lower mass loss, and, thus, lower reported adsorption capacity. This unusual procedure highlights the need for established protocol for mass measurement. For manometric systems that rely on *ex-situ* mass determination for a sample that is not oxygen-sensitive, but is hydrophilic like zeolite 13X, it is highly recommended to determine the wet mass in air, and then the dry mass immediately after outgassing and with nitrogen as the backfill gas to ensure similar buoyancy during the mass measurement. In order to avoid exposure to air during transfer as requested in the study protocol, a sample holder with air-tight seal to prevent water vapor in air from re-adsorbing onto the sample after outgassing is also recommended. Remeasurement using the recommended mass measurement protocol resolved the issue with DS17.

Within each dataset, the four isotherm measurements were generally reproducible, except for DS20, which had a large spread among all four isotherms. While other datasets (e.g. see DS2, DS3, DS4, DS8, DS10, DS19, DS21) exhibited slight differences in the adsorption isotherms between aliquots, the differences between the two aliquots in DS20 were significant (see Figure S4). Interestingly, DS20 also had the biggest difference in sample mass loss (22.5% vs. 20.2%) between its two aliquots. The participant also noted that the samples were not re-outgassed before the 2nd cycle measurements, which could account for the systematic drop in CO_2_ uptake in the 2nd cycle isotherms since CO_2_ or other adsorbed gases could still be present at the end of the desorption of the 1st cycle. Due to the irreproducibility between aliquots as well as cycles arising from mass variation and failure to outgas the samples in the 2nd cycle measurements, DS20 was determined to be unsuitable for inclusion in the determination of the reference isotherm.

For DS1, the participant used a new instrument and reported a slightly lower uptake at the 100 kPa region of the adsorption isotherms versus a second dataset measured using a separate instrument and submitted by the participant. This issue was later resolved by re-spanning the 100 kPa pressure transducer and recalibrating the manifold volume.

The blank isotherms from all datasets exhibited CO_2_ uptake of less than 0.015 mmol at 100 kPa (see Figure S11). Nevertheless, the effect of the blank can be quite large, especially if a smaller sample size is used. The effect of the blank subtraction for each dataset can be visualized in Figure S12 after normalizing the CO_2_ adsorption with the average sample mass used in the corresponding sample measurements. In particular, the blank affected the data by up to 0.11 mmol/g and by more than 0.02 mmol/g for DS3, DS8, DS10, DS11, DS16, and DS19, all of whom except DS19 applied the blank subtraction to their submitted data. For DS19, the blank subtraction would lower the adsorbed amount by 0.047 mmol/g at 100 kPa. Furthermore, the variation in mass loss between its two aliquots was just shy of that reported by DS20 (see earlier discussion). The participant (DS19) noted that in contrast to aliquot 1, aliquot 2 remained under vacuum at room temperature for several days after outgassing at 350 °C before starting the isotherm measurement. The much lower mass loss for aliquot 1 compared to aliquot 2 correlated with a lower CO_2_ uptake for aliquot 1. As such, DS19 was also deemed unsuitable for inclusion in the determination of the reference isotherm.

In total, the review of the submitted datasets identified 5 datasets (DS1, DS9, DS17, DS19, and DS20) for omission with identified measurement/data processing issues, such as inaccurate mass measurement, not performing blank subtraction, and not re-outgassing between cycles. Issues associated with mass, pressure, and volume measurements indicate care should be taken to ensure accurate system calibrations, as well as employing a sound protocol for mass measurements. For the interested reader, a summary of improper procedure can be found in Table S1 and a summary of the omitted datasets along with their identified origin of deviation can be found in Table S2.

### Reference low-pressure CO_2_ adsorption isotherm

**Fig. 2:**
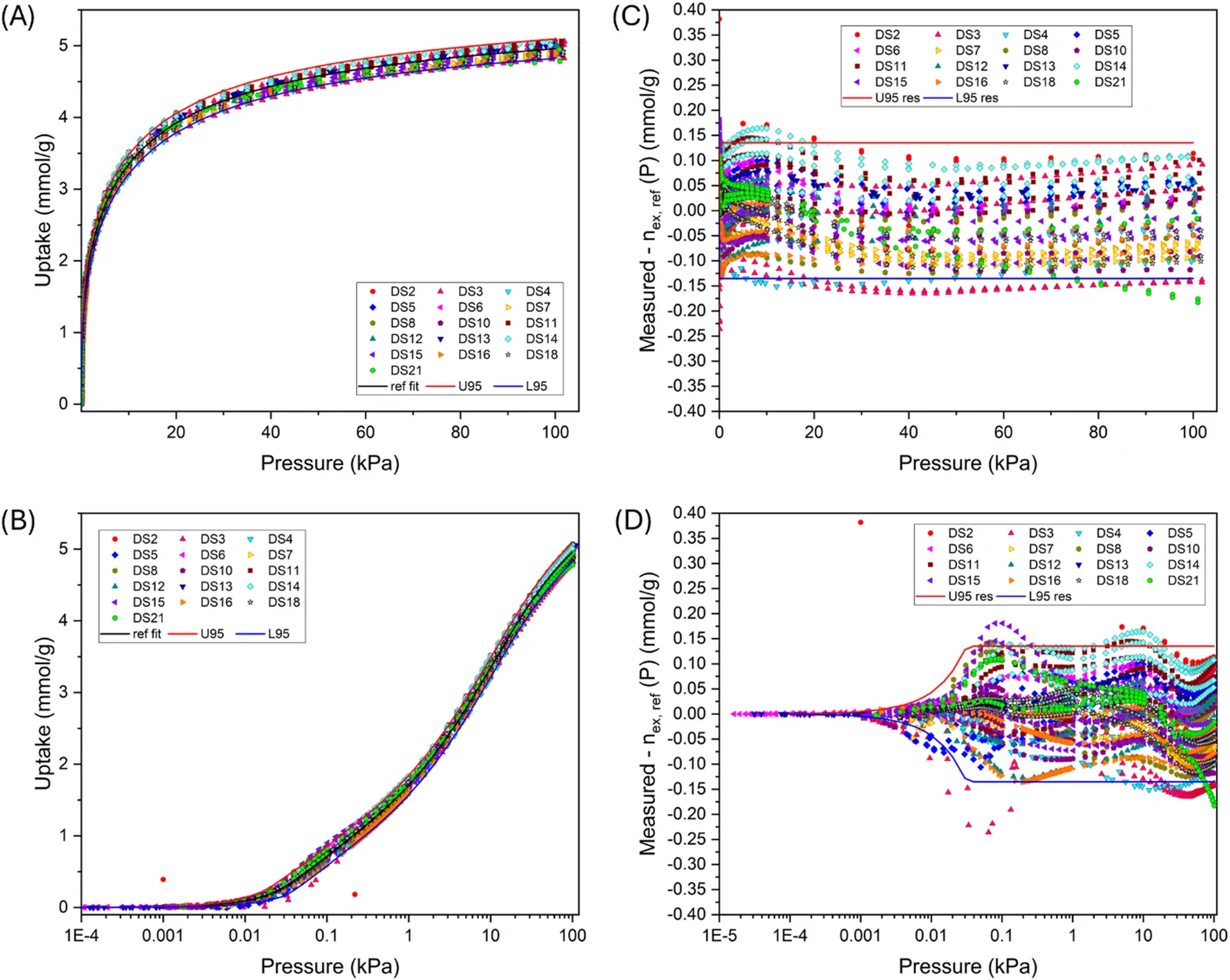
(**A**-**B**) Final surface excess CO_2_ adsorption isotherms at 25 °C for zeolite 13X used to determine the reference isotherm, along with the reference isotherm itself and the 95% uncertainty interval in (top) linear and (bottom) semi-log scales. (**C**-**D**) Residuals for the included datasets (Measured – *n*_*ex, ref*_ (*P*)) in units of mmol/g are shown on the right. For relative residuals (%) from predicted values, see Figure S14

Ultimately, 16 of the 21 datasets were used in the determination of the reference isotherm and are shown in Fig. 2. An empirical surface excess reference function was determined by optimizing the fit of Eq. (1) for *P* ≤ 0.0316 kPa and Eq. (2) for 0.0316 kPa ≤ *P* ≤ 100 kPa to the final remaining datasets and is also shown in Fig. 2. The optimized parameters are given in Table 2. Together the functions are predictive up to 100 kPa and have expanded uncertainty, U_k=2_, for the surface excess uptake given by (0.000350 + 4.2651*P/*kPa) mmol/g for *P* ≤ 0.0316 kPa, and 0.1352 mmol/g for 0.0316 kPa ≤ *P* ≤ 100 kPa. The predicted uptake and uncertainty values at select equilibrium pressure points are also tabulated in Table S3 in the Supplementary Information. The datasets and the reference adsorption isotherm are available through the NIST Database of Novel and Emerging Adsorbent Materials [21] at https://adsorption.nist.gov/isodb/index.php?DOI=10.1007/s10450-026-00701-3#biblio. For the interested reader, the sources of uncertainty based on contribution from between cycle, between aliquot, between lab, and measurement variability for 0.0316 kPa ≤ *P* ≤ 100 kPa can be found in Table 3. From Table 3, the smallest variability is observed between cycles (i.e., repeat measurements from one instrument, all else constant), indicating that measurements from each instrument or analysis station are generally repeatable. The variability between aliquots from each dataset is the largest, attributed to mass measurement uncertainty for the different aliquots, and for some datasets, potentially mixed with variation from different analysis stations used for different aliquots. The measurement (residual, fit) and between lab variability contribute to the reminding uncertainty.

The rationale for segmenting the fit with two functions was to better capture the behavior at low adsorption surface coverage where Henry’s Law is expected to apply. Henry’s Law predicts that at low adsorption coverage, the adsorbate adsorbed amount, *n*, is proportional to its partial pressure in the gas phase, *P*; i.e., *n* = *kP*, where *k* is Henry’s constant [22]. When log (*n*/(mmol/g)) is plotted against log (*P/*kPa), a linear fit is expected to have a slope of 1. From Eq. (1), the slope of such fit to final datasets is given by *b* = 1.027, which indicates that overall, these measured isotherms closely follow Henry’s Law below 0.0316 kPa.

To the right of the isotherms in Fig. 2 are their uptake residuals (measured isotherm minus reference isotherm), along with *U*_*k=2*_. The residuals show that the reference function sufficiently represents the final set of CO_2_/zeolite 13X adsorption isotherms over the full pressure range of the study.

Figure S13 shows the final datasets plotted in the log-log scale, which magnifies differences at the ultra-low pressures. Notable is DS2, whose two first measurement points deviate substantially from the reference values. This could be attributed to the generally high-pressure nature of the instrument used to collect DS2, which may not be able to record the pressures below 1 kPa accurately since the instrument is equipped with a 2-MPa pressure transducer with an accuracy of ± 1 kPa. It is also interesting to note that the first pressure points in some of the measurements (e.g. DS5, DS7, DS10) using dedicated low-pressure instruments also appear to lag in uptake slightly. A longer pre-analysis evacuation time and/or stricter equilibration criteria (e.g. longer time, or smaller change in pressure or mass over a given time interval) for the first few points could potentially help alleviate this issue. This might also help with aliquot 1 of DS3, which appears to have a significant number of data points outside of the 95% uncertainty bounds below 0.1 kPa if the issue was not with the pressure transducer itself. Lastly, aliquot 2 of DS3 also appears to deviate slightly above 15 kPa. While DS3 has deviation from the consensus mean outside the 95% uncertainty interval, it did not meet earlier criteria for data exclusion. Thus, delineating the cause for the variation in DS3 without extensive remeasurement is not possible. Some suspected sources for the deviation for aliquot 2 of DS3 include issues with mass measurement or the calibration of the 100 kPa pressure transducer used. Given that many dedicated low-pressure instruments are equipped with multiple pressure transducers to cover various pressure ranges (common ranges are 0.0133 kPa (or 0.133 kPa), 13.3 kPa, and 133 kPa), it is possible for a measurement to be accurate in one pressure range but inaccurate in another (e.g. see omitted DS1). Calibration of pressure transducers at intervals recommended by instrument manufacturers can minimize this issue. Furthermore, many of these routine instruments have multiple analysis stations, each with its own pressure transducers and manifold volume, highlighting the need to validate each station independently, along with application of the corresponding blank for each station.

## Recommendations

The protocol to use RGTM 10257 and the associated low-pressure reference CO_2_ adsorption isotherm up to 100 kPa at 25 °C are provided in Section S7 in the Supplementary Information. Moreover, the following recommendations and checklist for measuring this low-pressure CO_2_ adsorption isotherm are offered based on pitfalls identified in this work and building on recommendations from ILS1 [10]:

- ***T***, ***P***, **and** ***V***. Ensure good control and measurement of temperature (*T*), pressure (*P*), and volume (*V*), as these are important in accurate determination of the uptake. Thermocouples, pressure transducers, and reference manifold volume should be calibrated at intervals recommended by manufacturers to ensure accuracy. Keep measurements within the accurate measurable pressure range of the instrument by checking pressure transducer range and accuracy specifications, and critically questioning measurements outside the range.
- **Sample activation.** Complete sample activation is crucial, and for zeolite 13X, this involves outgassing at high-temperature and high-vacuum. Zeolite 13X must be activated at 350 °C for at least 12 h under high vacuum (≤ 1 cPa) to realize the reported reference isotherm. For gravimetric systems, activation is generally done in-situ. However, for many manometric systems, sample activation is typically done *ex-situ* and exposure to air and moisture must be avoided during handling of sample for weighing and transferring to analysis station. For *ex-situ* activation, a sample holder with air-tight seal is recommended. The average % mass loss from outgassing for RGTM 10257 observed in this study is 21.4 % using fresh sample. If the sample is reused in subsequent measurement cycles, the sample must be re-outgassed to achieve the reference isotherm.
- **Sample mass.** Determination of accurate sample mass is crucial for accurate determination of uptake. Sample should be completely activated. For manometric systems that rely on *ex-situ* mass determination for a sample that is not oxygen-sensitive, but is hydrophilic like zeolite 13X, it is highly recommended to determine the wet mass in air, and then the dry mass immediately after outgassing and with nitrogen as the backfill gas to ensure similar buoyancy during the mass measurement. A sample holder with air-tight seal is also recommended to prevent water vapor in air from re-adsorbing onto the sample after outgassing and during transfer. The sample mass to be used should be optimized to attain the best signal-to-noise ratio, according to the experimental parameters of the instrument being used. Typically, using a larger sample mass can help reduce relative uncertainty from mass measurements.
- **Sample volume determination/buoyancy correction/void volume correction**. Proper determination of sample volume is needed both for buoyancy correction in a gravimetric system, as well as void volume determination in a volumetric system. A buoyancy correction must be applied when using a gravimetric system, which is analogous to the use of void volume in a volumetric instrument to determine surface excess uptake. A skeletal density of ≈2.476 g/cm^3^ should be used for RGTM 10257 if required for measurement or data analysis.
- **Equation of state**. Identify the equation of state used to calculate fluid density, and use critically evaluated equations. The Span and Wagner equation of state is recommended for CO_2_ adsorption at 25 °C.
- **Blank correction**: A blank run subtraction corrects for uncompensated, experimental effects and should be performed whenever possible, and especially if the gas uptake for the blank (in mmol or mmol/g once normalized to the sample mass used in the sample measurement), is large relative to that for the sample. The blank adsorption isotherm should be measured in the same analysis station using the same sample holder and under the same experimental conditions as that used for the sample measurement.

## Conclusions and outlook

This interlaboratory study provides reference surface excess isotherm data for low-pressure CO_2_ adsorption on zeolite 13X (NIST RGTM 10257) at 25 °C from 1 Pa up to 100 kPa. The CO_2_/zeolite 13X reference data should prove useful for researchers interested in working with very low concentrations of CO_2_. The study demonstrated the importance of accurate mass determination, and routine calibrations of pressure transducers and manifold volumes, as many instruments used in this ILS are routine low-pressure manometric systems found in many labs. The reference isotherm has associated uptake uncertainties, *U*_*k=2*,_ of ≤ 0.136 mmol/g (or ≈ 3% relative uptake at 100 kPa). With the low overall uncertainty and generally reproducible results within each lab and between labs, we propose to develop RGTM 10257 (zeolite 13X) into a reference material. Results from ILS4 will be used to support the advancement of RGTM 10257 to NIST Reference Material status, which will not only provide a different zeolite, but also the first beaded-form addition to the existing NIST zeolitic reference materials. Lastly, the beaded form of RGTM 10257 offers the possibility of moving future ILSs to systems, such as breakthrough instruments that may require the sample to be in pelletized, beaded, or granular form. Plans are underway to utilize RGTM 10257 in a forthcoming interlaboratory study looking at multicomponent adsorption from breakthrough measurements.

**Table 1:** Experimental parameters of the participants

Dataset	Measurement method	Gas purity (%)	Sample size (g)	Outgas condition	Sample handling/weighing and transfer
1	Manometric, static	CO_2_: 99.999	0.9045, 0.8819	Outgassed at 350 °C for at least 12 h using a turbomolecular pump down to 5 × 10^− 2^ Pa. Samples re-outgassed between 1st and 2nd cycle.	Activated ex-situ in sealed sample cell, back-filled with N_2_ to determine dry mass, and transferred to instrument without exposure to air.
2	Gravimetric, static	CO_2_: 99.995	0.072, 0.068	Outgassed at 350 °C for at least 12 h using a turbomolecular pump down to 36 Pa (as measured by 2000 kPa PT)	Activated in-situ
3	Manometric, static	CO_2_: 99.999	0.1431, 0.132	Outgassed at 350 °C for at least 12 h using a turbomolecular pump down to 1 × 10^− 3^ Pa	Activated ex-situ in sealed sample cell, back-filled with N_2_ to determine dry mass, and transferred to instrument without exposure to air.
4	Manometric, static	CO_2_: 99.999	0.8689, 0.9370	Outgassed at 350 °C for at least 12 h using a turbomolecular pump (rated to 5 × 10^− 8^ Pa)	Activated ex-situ, removed for weighing, then loaded into instrument and reactivated in-situ.
5	Manometric, static	CO_2_: 99.9	0.085, 0.083	Outgassed at 350 °C for at least 12 h using a turbomolecular pump down to 5.6 × 10^− 3^ Pa	Activated in-situ in sealed sample cell, back-filled with N_2_ to determine dry mass, and reattached to instrument without exposure to air.
6	Manometric, static	He: 99.99995 CO_2_: 99.99995	0.1659, 0.1619	Outgassed at 350 °C for at least 12 h using a turbomolecular pump down to < 0.1 Pa	Activated in-situ, backfilled with N_2_ after pretreatment and measurements to determine dry sample mass.
7	Manometric, static	CO_2_: 99.999	0.8616, 0.8114, 0.8952, 0.9273 (4 aliquots)	Outgassed at 350 °C for at least 12 h using a turbomolecular pump down to 1 × 10^− 3^ Pa	Activated ex-situ in sealed sample cell, back-filled with N_2_ to determine dry mass, and transferred to instrument without exposure to air.
8	Gravimetric, dynamic vac	CO_2_: 99.999	0.02234, 0.0227	Outgassed at 350 °C for at least 12 h using a turbomolecular pump down to 3 × 10^− 3^ Pa	Activated in-situ
9	Manometric, static	CO_2_: 99.999	0.2386, 0.2076	Outgassed at 350 °C for at least 12 h using a turbomolecular pump	Activated ex-situ first. Tube was then weighed and activated in-situ with the same port as measurement.
10	Manometric, static	CO_2_: 99.999	0.1708, 0.1596	Outgassed at 350 °C for at least 12 h using a turbomolecular pump	Activated ex-situ first. Tube back-filled with nitrogen and very quickly measured and put back under vacuum. The exposure was limited.
11	Manometric, static	CO_2_: 99.995	0.1905, 0.1980	First activation heated at a rate of 1 °C/min to 350 °C and held at that temperature for 12 h under high vacuum with continuous pumping to 0.267 Pa. Heating for an additional 2 h at 350 °C between the 1st and 2nd measurement.	Activated in-situ. Sample tube with seal check was backfilled with N_2_ after pretreatment to determine dry mass and transferred to back instrument and re-evacuated using turbo pump.
12	Manometric, static	CO_2_: 99.999	0.2683, 0.3040	Outgassed at 350 °C for at least 12 h using a turbomolecular pump down to 2.67 × 10^− 3^ Pa	Activated in-situ, sample mass determined after outgassing.
13	Manometric, static	He: 99.999, CO_2_: 99.999	0.5277, 0.7523	Outgassed at 350 °C for at least 12 h using a turbomolecular pump	Activated in-situ, sample mass determined after measurement.
14	Gravimetric, static	CO_2_: 99.9993	0.039834, 0.025113	Outgassed at 350 °C for 12 h using a turbomolecular pump (10^− 5^ Pa) down to 10^− 4^ Pa	Activated in-situ
15	Manometric, static	CO_2_: 99.9993	0.0519, 0.1281	Outgassed at 350 °C for 12 h using a turbomolecular pump (10^− 5^ Pa) down to 10^− 4^ Pa	Activated ex-situ in sealed sample cell, back-filled with N_2_ to determine dry mass, and transferred to instrument without exposure to air.
16	Manometric, static	CO_2_: 99.995	0.1635, 0.1606	Outgassed at 350 °C for at least 12 h using a turbomolecular pump down to 2 × 10^− 3^ Pa	Activated ex-situ in sealed sample cell, back-filled with N_2_ to determine dry mass, and transferred to instrument without exposure to air.
17	Manometric, static	CO_2_: 99.999	0.4607, 0.5259	Outgassed at 350 °C for 12 h using a turbomolecular pump down to 9.9 × 10^− 4^ Pa	Activated in-situ. Sample cell assembly includes a glass filler rod to reduce void volume and a spring-loaded filter to avoid sample elutriation and air diffusion during sample weighing after activation. Sample backfilled with CO_2_ for weighing.
18	Manometric, static	CO_2_: 99.995	0.200, 0.193	Outgassed at 350 °C for 12 h using a turbomolecular pump down to 1.2 × 10^− 4^ Pa	Activated in-situ. After activation, the sample cell was purged with He, sealed and weighed to determine the mass after activation to avoid exposure to air as much as possible
19	Manometric, static	CO_2_: 99.9998	0.1008, 0.0984	Outgassed in vacuum at RT, then heated at 1 °C/min to 350 °C and held at 350 °C for 12 h under high vacuum down to 6.7 × 10^− 3^ Pa.	Activated ex-situ. The sample cell without and with sample after activation were both purged with He, sealed, and weighed to determine the sample mass.
20	Manometric, static	CO_2_: 99.999	0.0948, 0.1078	Outgassed at 350 °C for 12 h using a turbomolecular pump down to 3 × 10⁻³ Pa. Sample was not reoutgassed between cycles.	Activated ex-situ with sealed sample holder to avoid exposure to air. Dry mass determined after 2nd cycle isotherm.
21	Manometric, static	CO_2_: 99.9995	0.14498, 0.14550	Outgassed in vacuum at RT, then heated at 1 °C/min to 110 °C, and held at 110 °C for 2 h, then heated at 1 °C /min to 350 °C and held at 350 °C for 6 h to final vacuum of 10^− 5^ Pa. Repeated on analysis station after transfer. Sample was reoutgassed on analysis station between cycles.	The sample was stored at 75% RH. Activated *ex-situ*, and then in-situ in analysis port. Mass loss of two aliquots determined from thermogravimetric analysis (TGA) profile.

Dataset	Void volume/buoyancy correction	Equation of state	Temperature and stability	Balance resolution and stability	Pressure transducer accuracy	Blank correction
1	Void volume was determined using He expansion before isotherm measurement.	CO_2_: Virial equation	(25 ± 0.03) °C	0.1 mg, ± 0.2 mg	13.3 Pa: ± 0.15% reading 1.33 KPa: ± 0.15% reading 207 kPa: ± 0.065% F.S.	Blank measured, not applied
2	Buoyancy correction via sample volume determined from sample mass and ρ_skel_ = 2.476 g/cm^3^	CO_2_: Redlich-Kwong-Soave	(25 ± 0.02) °C	0.1 µg	2000 kPa: ± 0.05% F.S.	Blank not measured, not applied
3	Void volume was determined using He expansion.	CO_2_: Span and Wagner [20]	(25 ± 0.02) °C	0.1 mg	± 0.12% reading	Blank measured and applied
4	Void volume was determined using He expansion.	CO_2_: Virial equation	(25 ± 0.03) °C	0.1 mg, in 2.5 s	133 Pa: ± 0.15% reading 1.33 KPa: ± 0.15% reading 207 kPa: ± 0.15% F.S.	Blank measured, not applied
5	Void volume was determined using He expansion.	CO_2_: Span and Wagner	(25 ± 0.1) °C	0.1 mg; ± 0.1 mg	0–13.3 Pa: ±0.15% reading 1.33 kPa — 133 kPa 0.12% reading	Blank measured and applied
6	Void volume was determined using He expansion.	CO_2_: Span and Wagner	(25 ± 0.05) °C	0.1 mg, ± 0.1 mg	13.3 Pa: ± 0.25% reading 1.33 kPa: ± 0.5% reading 133 kPa: ± 0.15% F.S.	Blank measured, n_ex_ <0.002 mmol, not applied
7	Void volume was determined using He expansion before isotherm measurement.	CO_2_: Ideal gas with temperature-based non-ideality factor (1/torr)	(25 ± 0.1) °C	0.1 mg, ± 0.2 mg	13.3 Pa: ± 0.15% reading 1.33 KPa: ± 0.2% reading 133 kPa: ± 0.11% F.S.	Blank measured and applied
8	Buoyancy correction via sample volume determined from sample mass and ρ_skel_ = 2.476 g/cm^3^	CO_2_: Span and Wagner	(25 ± 0.2) °C	0.1 µg, < 0.3 µg	± 0.5% reading	Blank measured and applied
9	Void volume was determined using He expansion.	CO_2_: Ideal gas law	25 °C	0.1 mg, ± 0.1 mg	133 Pa: ± 0.15% reading 1.33 kPa: ± 0.12% reading 133 kPa: ± 0.12% reading	Blank measured and applied
10	Void volume was determined using He expansion.	CO_2_: Ideal gas law used with non-ideality factor	25 °C	0.1 mg, ± 0.1 mg	133 Pa: ± 0.15% reading 1.33 kPa: ± 0.15% reading 133 kPa: ± 0.11% F.S.	Blank measured and applied
11	Void volume was determined using He expansion	CO_2_: Span and Wagner	(25 ± 0.1) °C	0.1 mg, ± 0.1 mg	13.3 Pa: ± 0.15% reading 1.33 kPa: ± 0.12% reading 133 kPa: ± 0.12% reading	Blank measured and applied
12	Void volume was determined using He expansion	CO_2_: Span and Wagner	(25 ± 0.05) °C	0.1 mg, ± 0.1 mg	± 0.12% reading	Blank measured and applied
13	Void volume was determined using He expansion after isotherm measurement.	CO_2_: Span and Wagner	(25 ± 0.1) °C	0.1 mg, ± 0.1 mg	13.3 Pa: ± 0.25% reading 1.33 kPa: ± 0.5% reading 133 kPa: ± 0.15% F.S.	Blank measured < 0.02% n_ex_ sample, not applied
14	Buoyancy correction via sample volume determined from sample mass and ρ_skel_ = 2.476 g/cm^3^	CO_2_: Span and Wagner	(25 ± 0.1) °C	0.1 µg, ± 0.1 µg (± 1 µg long term)	100 kPa: ± 0.05% F.S.	Blank measured on non-adsorbing pyrex glass, (*m* = 0.047 g, ρ_skel_ = 2.23 g/cm^3^) and applied
15	Void volume was determined using He expansion	CO_2_: Span and Wagner	(25 ± 0.1) °C	0.1 mg, ± 0.2 mg	0.133 kPa: ± 0.15% reading 133 kPa: ± 0.11% F.S.	Blank measured on non-adsorbing pyrex glass (*m* = 0.061 g, ρ_skel_ = 2.23 g/cm^3^), and applied
16	Void volume was determined using He expansion before isotherm measurement	CO_2_: Span and Wagner	(25 ± 0.01) °C	0.1 mg, ± 0.1 mg	13.3 Pa: ± 0.15% reading 1.33 kPa: ± 0.12% reading 133 kPa: ± 0.12% reading	Blank measured for each sample tube and applied
17	Void volume was determined using He expansion prior to the isotherm measurement.	CO_2_: Span and Wagner	(25 ± 0.01) °C	0.1 mg, ± 0.1 mg	13.3 Pa: ± 0.25% F.S. 1.33 kPa: ± 0.25% F.S. 133 kPa: ± 0.25% F.S.	Blank measured and applied
18	Free space determination: AFSM method	CO_2_: Span and Wagner	(24.9 ± 0.1) °C	1 mg, ± 1 mg	± 1% reading	Blank measured and applied
19	Void volume was determined using He expansion. The manifold volume was calibrated using a specific quartz glass rod.	CO_2_: Span and Wagner	(25 ± 0.02) °C	0.1 mg, ± 1 mg	13.3 Pa: ± 0.2% reading 1.33 kPa: ± 0.2% reading 133 kPa: ± 0.5% F.S.	Blank measured, not applied
20	Void volume was determined using He expansion	CO_2_: semi-empirical vendor library	(25 ± 0.01) °C	0.1 mg, ± 0.1 mg	13.3 Pa: ± 0.25% F.S. 1.33 kPa: ± 0.25% F.S. 133 kPa: ± 0.25% F.S.	Blank measured, not applied
21	Void volume was determined using He expansion before isotherm measurement	CO_2_: Ideal gas	(25 ± 0.2) °C	2 ng, < 10 µg baseline drift, Accuracy = ± 0.01% of reading	± 0.2% reading	Blank measured, not reported, not applied (negligible uptake)

**Table 2:** Empirical reference function parameters

	Value	Uncertainty
*a*	1.0225072	0.0073134
*b*	1.0273389	0.0033490
*c*	-0.7161604	0.0180529
*d*	0.3524402	0.0191226
*e*	-0.0837392	0.0072556
*f*	0.0073234	0.0009181

No physical meaning should be associated with these parameters or the empirical reference function itself. All parameters are unitless

**Table 3:** Relative contribution of uncertainty, *u*, for each level in the dataset

Source	Description	Magnitude of *u* (mmol/g)
Cycle	Between cycle variability	0.017982
Aliquot	Between aliquot variability	0.047551
Lab	Between lab variability	0.025000
Residual	Measurement variability	0.039072

## Supplementary Information

Below is the link to the electronic supplementary material.

Supplementary Material 1

## Data Availability

The datasets and the reference adsorption isotherm are available through the NIST Database of Novel and Emerging Adsorbent Materials.
