# Supplementary material for: A reference low-pressure CO2 adsorption isotherm for zeolite 13X: results of an interlaboratory study
Source: Adsorption (Boston). 2026 Aug 28;32(7):42. doi: 10.1007/s10450-026-00701-3 (PMC13525031; doi:10.1007/s10450-026-00701-3)
Supplement: Supplementary file 1 [file 10450_2026_701_MOESM1_ESM.docx]

Electronic Supplementary Information to accompany:

A Reference Low-Pressure CO_2_ Adsorption Isotherm for Zeolite 13X: Results of an Interlaboratory Study

Huong Giang T. Nguyen,^1*^ David Newton,^1^ Riaz Ahmad,^2^ Carsten Prinz,^3^ Mario Zorrilla-Valtierra,^4^ Krista S. Walton,^4^ Killian Gmyrek,^5^ Ronny Pini,^5^ Camille Petit,^5^ Marcus Lange,^6^ Jens Mӧllmer,^6^ Evelyn Fuhrmann,^7^ Katharina Volz,^7^ Garrett Taggart,^8^ Andreas Schreiber,^9^ Yuko Konishi,^10^ Kazuyuki Nakai,^10^ Christos Tampaxis,^11^ Theodore Steriotis,^11^ Hai Wang,^12^ Charles David Brewster,^13^ Mi Tian,^13^ Rajan Jagpal,^13^ Siyi Liu,^13^ George Neville,^13^ Sunanda Sain,^13^ Timothy Mays,^13^ Riccardo Rea,^14^ Enzo Mangano,^14^ Stefano Brandani,^14^ Maxwell Tsipoaka,^15^ Fateme Rezaei,^15^ Adrien Houzé,^16^ Nicolas Heymans,^16^ Guy De Weireld,^16^ Charles E. Holland,^17^ Armin D. Ebner,^17^ James A. Ritter^17^

*Corresponding author e-mail: huong.nguyen@nist.gov

^1^National Institute of Standards and Technology, Gaithersburg MD, USA

^2^Anton Paar QuantaTec Inc. Boynton Beach FL, USA

^3^Federal Institute for Materials Research and Testing, Berlin, Germany

^4^Georgia Institute of Technology, Atlanta GA, USA

^5^Imperial College London, London, UK

^6^Institut für Nichtklassische Chemie, Leipzig, Germany

^7^Micromeritics GmbH, Unterschleißheim, Germany

^8^Microtrac Inc., York, PA, USA

^9^Microtrac Retsch GmbH, Haan, Germany

^10^MicrotracBEL, Suminoe-ku, Osaka, Japan

^11^National Centre for Scientific Research “Demokritos”, Athens, Greece

^12^National Institute of Metrology, Beijing, China

^13^University of Bath, Bath, UK

^14^University of Edinburgh, Edinburgh, UK

^15^University of Miami, Coral Gables, FL, USA

^16^University of Mons, Mons, Belgium

^17^University of South Carolina, Columbia SC, USA

**Table of Contents**

| Cover sheet | S1 |
| --- | --- |
|  |  |
| Section S1: Individual CO_2_/zeolite 13X sorption isotherms at 25 °C for each dataset | S3 |
| - Figures S1-S4. Surface excess CO_2_/zeolite 13X sorption isotherms at 25 °C - Figures S5-S8. Surface excess CO_2_/zeolite 13X sorption isotherms at 25 °C in semi-log scale |  |
|  |  |
| Section S2: CO_2_/zeolite 13X adsorption isotherms at 25 °C with expanded range | S11 |
| - Figure S9. CO_2_/zeolite 13X adsorption isotherms at 25 °C extended range high-pressure (HP) data |  |
|  |  |
| Section S3: Supporting data analysis for determination of datasets for omission | S12 |
| - Figure S10. Zeolite 13X percent mass loss after outgassing. - Figure S11. Blank measurements of surface excess CO_2_ sorption isotherms at 25 °C. - Figure S12. Blank measurements of surface excess CO_2_ adsorption isotherms at 25 °C normalized against mass of sample used in (A) linear scale and (B) semi-log scale. - Table S1: Summary of improper procedures - Table S2. Summary of issues identified for omitted datasets |  |
|  |  |
| Section S4: Final datasets shown in log-log scale and relative residuals | S16 |
| - Figures S13. Final surface excess CO_2_ adsorption isotherms at 25 °C for zeolite 13X plotted in log-log scale - Figure S14. Residuals for the included datasets in % deviation in (A) linear scale and (B) semi-log scale. |  |
|  |  |
| Section S5: Omitted datasets’ residuals against reference data | S18 |
| - Figure S15. Surface excess CO_2_ adsorption isotherms at 25 °C for zeolite 13X for omitted datasets, reference isotherm, and residuals |  |
|  |  |
| Section S6: Remeasured or reprocessed omitted datasets | S19 |
| - Figure S16. Surface excess CO_2_ adsorption isotherms at 25 °C for zeolite 13X for remeasured or reprocessed omitted datasets, reference isotherm, and residuals |  |
|  |  |
| Section S6: Protocol to use RGTM 10257 (zeolite 13X) and low-pressure CO_2_ reference adsorption isotherm at 25 ºC. | S20 |
| - Protocol to use RGTM 10257 and low-pressure CO_2_ reference adsorption isotherm - Table S3. Reference CO_2_/zeolite 13X uptake at 25 ºC at select equilibrium pressures |  |

**Section S1: Individual CO_2_/zeolite 13X sorption isotherms at 25 °C for each dataset**

Figure S1. Surface excess CO_2_ sorption isotherms at 25 °C for zeolite 13X for DS1-DS6.

Figure S2. Surface excess CO_2_ sorption isotherms at 25 °C for zeolite 13X for DS7-DS12.

Figure S3. Surface excess CO_2_ sorption isotherms at 25 °C for zeolite 13X for DS13-DS18.

Figure S4. Surface excess CO_2_ sorption isotherms at 25 °C for zeolite 13X for DS19-DS21.

Figure S5. Surface excess CO_2_ sorption isotherms at 25 °C for zeolite 13X for DS1-DS6 in semi-log scale.

Figure S6. Surface excess CO_2_ sorption isotherms at 25 °C for zeolite 13X for DS7-DS12 in semi-log scale.

Figure S7. Surface excess CO_2_ sorption isotherms at 25 °C for zeolite 13X for DS13-DS18 in semi-log scale.

Figure S8. Surface excess CO_2_ sorption isotherms at 25 °C for zeolite 13X for DS19-DS21 in semi-log scale.

**Section S2: CO_2_/zeolite 13X isotherms at 25 °C with extended range**

Figure S9. Surface excess CO_2_ adsorption isotherms at 25 °C for zeolite 13X with extended range high-pressure (HP) data. P_0_ = 6.43 MPa using Span and Wagner equation of state.

**Section S3: Supporting data analysis for determination of datasets for omission**

Figure S10. Zeolite 13X percent mass loss after outgassing. Note that the percent mass loss could not be determined for DS12 as the wet sample mass was not provided. The reported zeolite mass prior to outgassing for aliquot 2 of DS2 appears to belong to a sample that may have already been previously outgassed, so their mass loss (< 3%) is not suitable for comparison here. DS21 reported that they subjected the sample to 75% RH for the “wet” sample mass. The average mass loss was determined along with the 95% uncertainty interval (2 standard deviations from the mean).

Figure S11. Blank measurements of surface excess CO_2_ sorption isotherms at 25 °C.

Figure S12. Blank measurements of surface excess CO_2_ adsorption isotherms at 25 °C normalized against mass of sample used in (A) linear scale and (B) semi-log scale.

Table S1: Summary of improper procedures

| **Dataset** | **Improper procedure** |
| --- | --- |
| DS17 | Dry mass measurement after back-filling with CO_2_ |
| DS19 | Not applying blank subtraction when blank is large |
| DS20 | Not re-outgassing sample before 2^nd^ cycle measurements |

Table S2. Summary of issues identified for omitted datasets

| **Dataset** | **Reason dataset was flagged** | **Inferred origin of deviation** | **What was done differently for remeasurement or reprocessing** |
| --- | --- | --- | --- |
| DS1 | Participant reported that DS1 is lower in comparison to another dataset they submitted | Calibration issue with 100 kPa pressure transducer and manifold volume ^a^ | Re-spanned 100 kPa pressure transducer followed by recalibration of manifold volume before remeasurement |
| DS9 | Slightly higher uptake than all the other datasets. This participant also submitted two datasets, and noted a difference in them. | Mass measurement issue ^b, c^ | Renormalized data to average zeolite 13X mass loss of the other dataset submitted by the participant |
| DS17 | Unusually low uptake | Mass measurement issue due to incorrect determination of dry mass ^a,^ ^c^ | Determined dry sample mass by backfilling with N_2_ instead of CO_2_ |
| DS19 | Did not perform blank subtraction | Large blank was not subtracted;^b, c^ Lower than average mass losses and noticeable variation in between aliquot 1 vs aliquot 2 potentially from outgassing issues ^b, c^ | Applied blank subtraction and renormalized data using average zeolite 13X mass loss from ILS4 |
| DS20 | Large variation between aliquots and between cycles | Mass measurement issue for variation between aliquots.^b, c^ Lack of re-outgassing for variation between cycles. ^c^ | Renormalized cyc1 data using average zeolite 13X mass loss from ILS4 |

*^a^* Origin of deviation supported based on remeasurement of data by participant

*^b^* Origin of deviation supported based on reprocessing of data by NIST

*^c^* Origin of deviation identified from evaluation of experimental data or procedure

**Section S4: Final datasets shown in log-log scale and relative residuals**

Figure S13. Final surface excess CO_2_ adsorption isotherms at 25 °C for zeolite 13X used to determine the reference isotherm, along with the reference isotherm itself and the 95% uncertainty interval shown in log-log scale.

Figure S14. Residuals for the included datasets ([(Measured – *n_ex,ref_* (*P*))/*n_ex,ref_* (*P*)]*100%) in % deviation in (A) linear scale and (B) semi-log scale.

**Section S5: Omitted datasets’ residuals against reference data**

Figure S15. (A-B) Surface excess CO_2_ adsorption isotherms at 25 °C for zeolite 13X for omitted datasets, along with the reference isotherm and the 95% uncertainty interval. In all cases, each outlying dataset has one or more isotherms with many datapoints that fall outside of the 95% uncertainty intervals. (C-D) Residuals for the outlying datasets (Measured – *n_ex,ref_* (*P*)) in units of mmol/g are shown on the right.

**Section S6: Remeasured or reprocessed omitted datasets**

Figure S16. (A-B) Surface excess CO_2_ adsorption isotherms at 25 °C for zeolite 13X for remeasured or reprocessed datasets, along with the reference isotherm and the 95% uncertainty interval. (C-D) Residuals for the remeasured or reprocessed datasets (Measured – *n_ex,ref_* (*P*)) in units of mmol/g are shown on the right. rm = remeasured; rp = reprocessed. See Table S2 for details on what was done differently for rm or rp datasets.

S**ection S7: Protocol to use RGTM 10257 (zeolite 13X) and low-pressure CO_2_ reference adsorption isotherm at 25 ºC. (Recommended practices for realizing the reference isotherm are provided in the manuscript.)**

Materials: The adsorbent is NIST reference grade test material 10257 (zeolite 13X). The sample mass to be used should be optimized to achieve the best signal-to-noise ratio, according to the experimental parameters of the instrument being used. The adsorptive, carbon dioxide, should have a purity of no less than 99.999%.

Sample pretreatment: The zeolite should be outgassed using a turbomolecular pump and slowly heated to 623 K (350 °C) (1 degree per minute, nominally) and held at that temperature for at least 12 hours under high vacuum (<10^-5^ kPa) with continuous pumping. If the outgassing is performed in a separate manifold, prevent contact with air when transferring to the analysis instrument.

Measurement of adsorption isotherms: Carbon dioxide adsorption isotherms should be measured at 298.15 K (25 °C) over a pressure range of 2 Pa to 100 kPa. The same measurement is also to be done without the adsorbent to perform a “blank correction.” This blank should be subtracted from the adsorption isotherm.^[1]^ The Span and Wagner^[2]^ equation of state is to be used to determine the density/compressibility of the gas for the determination of surface excess uptake. RGTM 10257 has a skeletal density of ≈2.476 g/cm^3^, which is to be used when performing buoyancy correction for gravimetric technique or void volume determination for volumetric technique.

Comparison to the empirical reference isotherm function: Measured isotherms should fall within the 95% uncertainty interval of the reference isotherm function given by Eq. (1) for *P* ≤ 0.0316 kPa, and Eq. (2) for 0.0316 kPa ≤ *P* ≤ 100 kPa. See Table S3 for tabulated reference data.

$\frac{n_{ex,ref}\left( P \right)}{(mmol/g)}={10}^{a+b \mathrm{lo}g_{10}(\frac{P}{k\mathrm{Pa}})}$	Eq. (1)

$\frac{n_{ex,ref}\left( P \right)}{(mmol/g )}={10}^{a+b \mathrm{lo}g_{10}\left( \frac{P}{\mathrm{kPa}} \right)+c \left( {{(log}_{10} \left( \frac{P}{\mathrm{kPa}} \right)+1.5)}^{2} \right) + d \left( {{(log}_{10} \left( \frac{P}{\mathrm{kPa}} \right)+1.5)}^{3} \right)+e \left( {{(log}_{10} \left( \frac{P}{\mathrm{kPa}} \right)+1.5)}^{4} \right)+f \left( {{(log}_{10} \left( \frac{P}{\mathrm{kPa}} \right)+1.5)}^{5} \right)}$	Eq. (2)

[*n_ex,ref_* = surface excess uptake (mmol/g), *P* = equilibrium pressure (kPa), *a =* 1.0225072 , *b =* 1.0273389, *c =* -0.7161604, *d =* 0.3524402, *e =* -0.0837392, and *f* = 0.0073234]

Together the functions are predictive up to 100 kPa and have expanded uncertainty, U_k=2_, for the surface excess uptake given by (0.000350 + 4.2651*P/*kPa) mmol/g for *P* ≤ 0.0316 kPa, and 0.1352 mmol/g for 0.0316 kPa ≤ *P* ≤ 100 kPa.

Table S3. Reference CO_2_/zeolite 13X uptake at 25 ºC at select equilibrium pressures

| Pressure  (kPa) | Uptake (mmol/g) | Upper 95% Bound (mmol/g) | Lower 95% Bound  (mmol/g) | Fit |
| --- | --- | --- | --- | --- |
| 0.001 | 0.009 | 0.013 | 0.004 | Eq.(1) |
| 0.002 | 0.018 | 0.027 | 0.009 | Eq.(1) |
| 0.004 | 0.036 | 0.054 | 0.019 | Eq.(1) |
| 0.006 | 0.055 | 0.081 | 0.029 | Eq.(1) |
| 0.008 | 0.074 | 0.108 | 0.039 | Eq.(1) |
| 0.01 | 0.093 | 0.136 | 0.050 | Eq.(1) |
| 0.015 | 0.141 | 0.205 | 0.077 | Eq.(1) |
| 0.02 | 0.189 | 0.275 | 0.104 | Eq.(1) |
| 0.03 | 0.287 | 0.415 | 0.159 | Eq.(1) |
| 0.04 | 0.380 | 0.515 | 0.244 | Eq. (2) |
| 0.05 | 0.457 | 0.593 | 0.322 | Eq. (2) |
| 0.06 | 0.524 | 0.659 | 0.388 | Eq. (2) |
| 0.07 | 0.581 | 0.716 | 0.446 | Eq. (2) |
| 0.08 | 0.631 | 0.766 | 0.496 | Eq. (2) |
| 0.09 | 0.676 | 0.811 | 0.541 | Eq. (2) |
| 0.1 | 0.716 | 0.852 | 0.581 | Eq. (2) |
| 0.15 | 0.873 | 1.009 | 0.738 | Eq. (2) |
| 0.2 | 0.986 | 1.121 | 0.851 | Eq. (2) |
| 0.3 | 1.150 | 1.285 | 1.014 | Eq. (2) |
| 0.4 | 1.271 | 1.406 | 1.135 | Eq. (2) |
| 0.5 | 1.369 | 1.504 | 1.233 | Eq. (2) |
| 0.6 | 1.452 | 1.587 | 1.317 | Eq. (2) |
| 0.7 | 1.526 | 1.661 | 1.391 | Eq. (2) |
| 0.8 | 1.592 | 1.727 | 1.457 | Eq. (2) |
| 0.9 | 1.652 | 1.787 | 1.517 | Eq. (2) |
| 1 | 1.707 | 1.843 | 1.572 | Eq. (2) |
| 1.5 | 1.937 | 2.072 | 1.801 | Eq. (2) |
| 2 | 2.116 | 2.251 | 1.980 | Eq. (2) |
| 3 | 2.392 | 2.527 | 2.257 | Eq. (2) |
| 4 | 2.604 | 2.740 | 2.469 | Eq. (2) |
| 5 | 2.778 | 2.913 | 2.643 | Eq. (2) |
| 6 | 2.924 | 3.059 | 2.789 | Eq. (2) |
| 7 | 3.050 | 3.186 | 2.915 | Eq. (2) |
| 8 | 3.161 | 3.296 | 3.026 | Eq. (2) |
| 9 | 3.260 | 3.395 | 3.125 | Eq. (2) |
| 10 | 3.348 | 3.484 | 3.213 | Eq. (2) |
| 12.5 | 3.536 | 3.671 | 3.401 | Eq. (2) |
| 15 | 3.688 | 3.824 | 3.553 | Eq. (2) |

Table S3. Reference CO_2_/zeolite 13X uptake at 25 ºC at select equilibrium pressures (continued)

| Pressure  (kPa) | Uptake (mmol/g) | Upper 95% Bound (mmol/g) | Lower 95% Bound  (mmol/g) | Fit |
| --- | --- | --- | --- | --- |
| 17.5 | 3.815 | 3.950 | 3.680 | Eq. (2) |
| 20 | 3.923 | 4.058 | 3.788 | Eq. (2) |
| 25 | 4.098 | 4.233 | 3.963 | Eq. (2) |
| 30 | 4.234 | 4.369 | 4.099 | Eq. (2) |
| 35 | 4.344 | 4.480 | 4.209 | Eq. (2) |
| 40 | 4.436 | 4.571 | 4.300 | Eq. (2) |
| 45 | 4.513 | 4.648 | 4.377 | Eq. (2) |
| 50 | 4.579 | 4.714 | 4.444 | Eq. (2) |
| 55 | 4.637 | 4.772 | 4.501 | Eq. (2) |
| 60 | 4.688 | 4.823 | 4.552 | Eq. (2) |
| 65 | 4.733 | 4.868 | 4.598 | Eq. (2) |
| 70 | 4.774 | 4.909 | 4.639 | Eq. (2) |
| 75 | 4.811 | 4.947 | 4.676 | Eq. (2) |
| 80 | 4.845 | 4.981 | 4.710 | Eq. (2) |
| 85 | 4.877 | 5.012 | 4.742 | Eq. (2) |
| 90 | 4.906 | 5.041 | 4.771 | Eq. (2) |
| 95 | 4.933 | 5.068 | 4.798 | Eq. (2) |
| 100 | 4.959 | 5.094 | 4.824 | Eq. (2) |

1. H. G. T. Nguyen, J. C. Horn, M. Thommes, R. D. van Zee, L. Espinal, Experimental Aspects of Buoyancy Correction in Measuring Reliable High-pressure Excess Adsorption Isotherms Using the Gravimetric Method, *Meas. Sci. Technol.* **2017**, *28*, 125802.
2. R. **Span**, W. **Wagner,** A New Equation of State for **Carbon Dioxide** Covering the Fluid Region from Triple-Point Temperature to 1100 K at Pressures up to 800 MPa, *J. Phys. Chem. Ref. Data*, **1996**, *25*, 1509-1597.
